# Factors associated with admission to bed-based care: observational prospective cohort study in a multidisciplinary same day emergency care unit (SDEC)

**DOI:** 10.1186/s12877-020-01942-9

**Published:** 2021-01-06

**Authors:** Tania C. N. Elias, Jordan Bowen, Royah Hassanzadeh, Daniel S. Lasserson, Sarah T. Pendlebury

**Affiliations:** 1 Wolfson Centre for Prevention of Stroke and Dementia, Nuffield Department of Clinical Neurosciences, John Radcliffe Hospital, and the University of Oxford, Wolfson Building, Oxford, OX3 9DU England; 2 Departments of Acute Internal Medicine and Geratology, Oxford University Hospitals NHS Foundation Trust, John Radcliffe Hospital, Oxford, OX3 9DU England; 3 PIONEER Health Data Research Hub, Institute for Applied Health Research, University of Birmingham, Birmingham, B15 2TT England; 4 Department of Acute Medicine, City Hospital, Sandwell and West Birmingham Hospitals NHS Trust, Birmingham, B18 7QH England; 5 NIHR Oxford Biomedical Research Centre, John Radcliffe Hospital, Oxford, OX3 9DU England

**Keywords:** Same day emergency care, Ambulatory care, Predictive factors, Hospital admission, Bed-based care, Frailty, Delirium, Multi-morbidity

## Abstract

**Background:**

The development of ambulatory emergency care services, now called ‘Same Day Emergency Care’ (SDEC) has been advocated to provide sustainable high quality healthcare in an ageing population. However, there are few data on SDEC and the factors associated with successful ambulatory care in frail older people. We therefore undertook a prospective observational study to determine i) the clinical characteristics and frailty burden of a cohort in an SDEC designed around the needs of older patients and ii) the factors associated with hospital admission within 30-days after initial assessment.

**Methods:**

The study setting was the multidisciplinary Abingdon Emergency Medical Unit (EMU) located in a community hospital and led by a senior interface physician (geriatrician or general practitioner). Consecutive patients from August–December 2015 were assessed using a structured paper proforma including cognitive/delirium screen, comorbidities, functional, social, and nutritional status. Physiologic parameters were recorded. Illness severity was quantified using the Systemic Inflammatory Response Syndrome (SIRS> 1). Factors associated with hospitalization within 30-days were determined using multivariable logistic regression.

**Results:**

Among 533 patients (median (IQR) age = 81 (68–87), 315 (59%) female), 453 (86%) were living at home but 283 (54%) required some form of care and 299 (56%) had Barthel< 20. Falls, urinary incontinence and dementia affected 81/189 (43%), 50 (26%) and 40 (21%) of those aged > 85 years.” Severe illness was present in 148 (28%) with broadly similar rates across age groups. Overall, 210 (39%) patients had a hospital admission within 30-days with higher rates in older patients: 96 (87%) of < 65 years remained on an ambulatory pathway versus only 91 (48%) of ≥ 85 years (*p* < 0.0001). Factors independently associated with hospital admission were severe illness (SIRS/point, OR = 1.46,95% CI = 1.15–1.87, *p* = 0.002) and markers of frailty: delirium (OR = 11.28,3.07–41.44, *p* < 0.0001), increased care needs (OR = 3.08,1.55–6.12, *p* = 0.001), transport requirement (OR = 1.92,1.13–3.27), and poor nutrition (OR = 1.13–3.79, *p* = 0.02).

**Conclusions:**

Even in an SDEC with a multidisciplinary approach, rates of hospital admission in those with severe illness and frailty were high. Further studies are required to understand the key components of hospital bed-based care that need to be replicated by models delivering acute frailty care closer to home, and the feasibility, cost-effectiveness and patient/carer acceptability of such models.

**Supplementary Information:**

The online version contains supplementary material available at 10.1186/s12877-020-01942-9.

## Background

Major restructuring of existing clinical services is required to provide sustainable high quality healthcare for increasing numbers of older people with complex co-morbidity [1–3]. In the United Kingdom, the Royal College of Physicians’ has called for an ‘ambulatory by default’ approach (Future Hospital Commission) [1] particularly for frail/older patients who are frequent users of acute internal medicine services, have high rates of hospital admission for conditions considered suitable for ambulatory care [3] and may be harmed by unnecessary in-patient stays [4, 5]. To achieve high quality care will require a multidisciplinary approach [6–8], incorporating comprehensive geriatric assessment [9] and NHS England has developed a support programme to encourage the development of ambulatory emergency care services, now called ‘Same Day Emergency Care’ (SDEC) [10, 11].

However, there are few data on SDEC and particularly on the factors associated with successful ambulatory care in older and co-morbid patients with frailty where hospitalization rates are high [12]. Studies of SDEC in younger cohorts or services dedicated to specific conditions (e.g. heart failure, pulmonary embolism, syncope) [12] may not be applicable to older patients in whom multi-morbidity, frailty and non-specific presentations of disease are common. Data from emergency departments/medical units suggest that SDEC is used less frequently in older, frail people [13]. Small studies suggest SDEC may reduce admissions in carefully selected older patients, although numbers are too small to draw firm conclusions regarding later readmission rates [14, 15], and the care model is liked by patients [16].

In the current study, we report data from a specialist-led community hospital-based SDEC unit with multidisciplinary team input and inclusive referral criteria, locally termed the ambulatory emergency multidisciplinary unit (EMU). The EMU was set up with the specific aim to provide ambulatory emergency care focused on the needs of unselected older adults with acute medical illness. Using data from a consecutive cohort of predominantly older patients prospectively assessed in the EMU, we determined i) the case-mix and frailty burden of the cohort and ii) the factors associated with any use of bed-based care (any hospital admission) up to 30 days after initial EMU assessment.

## Methods

### Setting

The EMU was set up in 2010 following a commitment by the Oxford University Hospitals NHS Foundation Trust (OUHFT) to develop ambulatory emergency care pathways for older people. The service subsequently won the Guardian Healthcare Innovation Award in 2013 [17]. Although the EMU was designed to be responsive to the needs of frail older patients, it is not exclusively for older patients and younger people may also be referred to the service. The EMU is based in Abingdon Community Hospital and includes six in-patient beds for EMU patients requiring hospital admission from a catchment population of approximately 140,000. The regional district general hospital services including the emergency department are located in the OUHFT approximately 10 miles away. The EMU comprises six assessment cubicles and two rooms and is staffed 7 days a week by a consultant geriatrician or final year trainee geriatrician, or experienced general practitioner (GP) with skills in acute medicine and geriatric medicine (locally termed ‘Senior Interface Physician’), a primary care trainee doctor, a nursing team (of advanced practitioners, staff nurses and associate practitioners), an occupational therapist, a physiotherapist and a social worker.

Emergency referrals of adult medical patients are made by primary care physicians, paramedics or local acute in-patient services (patients cannot self-present to the service). Local primary care clinicians and ambulance crews were made aware of the service by written information and regular educational sessions organised by a primary care physician working in both EMU and a local primary care practice. Referrers call directly through to the senior clinician or nurse, and patients are then seen on the same day, given a future appointment, directed to an alternative service, or treated in the community without EMU attendance. There are no absolute exclusions to EMU assessment with patient-centred decisions being made for each individual. However, patients with high degrees of acuity (severe physiologic instability, acute coronary syndromes, acute stroke, trauma, surgical emergencies) without advance care plans are routinely redirected to the acute hospital. At the time of this study, a dedicated EMU patient transport service was available if required.

Point-of-care blood testing provides results within minutes (electrolytes, creatinine, haemoglobin, C-reactive protein, troponin, lactate, blood gases, prothrombin time). Additional laboratory-based tests are sent for analysis to the OUHFT (results accessible within 24 h). Plain x-ray is available on site. For additional imaging (e.g. computed tomography, ultrasound), slots are arranged at the OUHFT. Intravenous treatments (including blood products) can be administered in EMU and there is a wide stock of oral medications, supplemented by access to a nearby pharmacy by prescription. Equipment to aid functional ability can be provided by therapists and a dedicated social worker is available to facilitate discharge-planning. Resuscitation equipment is available for advanced adult life support.

After assessment in EMU, patients may be discharged (with or without further review in EMU or elsewhere), referred for domiciliary treatment by the ‘Hospital at Home’ nursing team (who are able to administer intravenous treatments and carry out phlebotomy) or admitted to a hospital bed in the Abingdon Hospital or the OUHFT.

### Patient cohort

All consecutive patients referred for assessment in EMU between August–December 2015 were included in the current study. There were no exclusion criteria. All data were routinely acquired as part of standard patient care and anonymized data were entered into the Oxford Cognitive Co-morbidity and Frailty Ageing Research Database (ORCHARD). ORCHARD was specifically set-up to inform the care of older and frail patients and has a patient and public involvement group including older patients and carers which informs study planning and design [18]. The local research ethics committee (REC reference 18/SC/0184) has approved the use of ORCHARD data for research purposes waiving the need for informed consent, and the agreement of the OUHFT Divisional Management was also obtained for this substudy (Datix Number 3812).

Patients were prospectively assessed using a structured paper clerking proforma to ensure systematic and standardized recording of the history and examination findings as described previously [19], by clinicians working in EMU (JB, TE, RH). The clerking proforma includes a cognitive screen with the Confusion Assessment Method (CAM) [20], cognitive test (the abbreviated mental test score (AMTS) [21]) and documentation of diagnosed dementia and delirium, validated for use in the emergency medical setting [22]. Demographic data, presenting complaint, past medical and drug history, living arrangements, transport requirements, care needs (‘care’ was defined as regular (from weekly to 24-hourly) help with instrumental (e.g. shopping or cleaning) or personal (e.g. washing or dressing) activities of daily living), and number of comorbidities were recorded from the patient, relatives or carers, and GP and medical records. The Charlson index for comorbidities was calculated [23]. Barthel Index of Activities of Daily Living [24] and modified Rankin Scale (mRS) [25] were recorded for level of functional dependence. The Braden Scale [26], to quantify pressure ulcer risk, and the Malnutrition Universal Screening Tool (MUST) [27] were recorded by nursing staff.

Although no specific frailty scale/score was measured, we used data from the comprehensive geriatric assessment undertaken as part of the EMU assessment to provide proxy measures or “markers of frailty”. Markers of frailty included reduced function (need for care, history of falls, decline in mobility), visual or hearing impairment, incontinence, poor nutrition (MUST score), pressure sore risk, and impaired cognition (delirium, dementia, AMTS score). Clinicians were also asked to answer the following questions: “Do you judge the patient to be frail?”, and “Do you judge the patient to be clinically dehydrated?”

Physiological parameters on admission (pulse, temperature, systolic and diastolic blood pressure, oxygen saturation and respiratory rate) were taken from the patient chart. Illness severity was quantified using the Systemic Inflammatory Response Syndrome (SIRS) score [28] and National Early Warning Score (NEWS) [29] as these require only routinely collected clinical data. Severe illness was defined as SIRS> 1, and NEWS> 4. Anaemia was diagnosed when both haemoglobin level was less than the local laboratory reference range and this was relevant to the primary presenting complaint. Acute kidney injury (AKI) was defined as Stage 1 or above of the Acute Kidney Injury Network (AKIN) Classification [30].

### Outcomes

Ambulatory care status was defined as living at home vs any hospital admission, within 30 days from the first assessment in EMU. EMU patients requiring acute hospital admission are admitted either to the dedicated in-patient beds at Abingdon community hospital or to the OUHFT acute hospital in-patient services. Ambulatory status was recorded by the clinical researchers embedded in EMU (JB, TE, RH) supplemented by hand-searching of EMU paper and electronic records and OUHFT electronic records. 30-day ambulatory outcome data were unavailable for 8 patients (5 aged < 65 years, 2 aged 65–84 years and 1 aged ≥ 85 years). Mortality data was obtained from EMU and OUHFT electronic records with follow-up for death to 1 year. Mortality data were unavailable for 12 patients who were normally resident outside the region.

### Statistical analyses

Any patient re-referred with a new illness episode during the study was included as a separate new case, but the first illness episode only was used for mortality analyses. Missing data were < 5% for all variables (see Additional file 1: Table 1). Missing data were not imputed except for white cell count (not always done in relatively well patients) and AMTS which were imputed as normal.

Differences in clinical characteristics across age groups, defined as < 65, 65–84 and ≥ 85 years, were compared using ANOVA for continuous variables and chi square for categorical variables. Associations between potential predictive factors and any bed-based care at 30 day follow up were determined by binary logistic regression to generate odds ratios adjusted for age and sex. In view of the number of potentially important co-variables associated with admission to bed based care after an initial assessment in the ambulatory setting, we highlighted those variables significant at *p* < 0.001 i.e. those variables that remained significant after adopting a Bonferroni correction. We also compared univariable associates of admission adjusted for age and sex between patients with immediate hospital admission from EMU with those admitted later within the 30-days.

To determine the independent associates of any bed-based care, univariable associates significant at the *p* < 0.1 level were entered into a multivariable logistic regression model with forward selection. The SIRS and NEWS were each entered into the models separately. Items not routinely measured in most acute care services at the point of first patient assessment (MUST, Braden, mRS, Barthel, and Charlson index scores) were omitted from the primary analyses but included in additional analyses presented in the Additional file 1. Models were run with and without inclusion of patients with delirium to determine the factors associated with admission in non-delirious patients since delirium was strongly associated with admission. Prior to modelling, variables were assessed for collinearity (tolerance statistic < 0.4), and all had tolerances of > 0.5. Statistical analyses were performed using SPSS version 25.

## Results

**Fig. 1:**
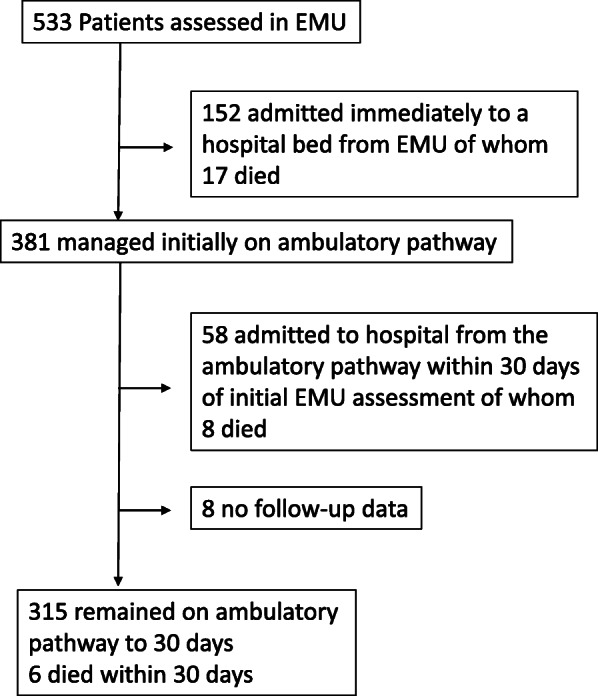
Flow diagram showing the patient cohort and the number admitted immediately from EMU, the number initially managed on an ambulatory pathway, the number admitted after initial EMU assessment but within 30-days, and the number remaining on an ambulatory pathway at 30-days

Five hundred and thirty three consecutive new patient referrals (mean/SD age = 75.0/17.5 years, median (IQR) age = 81 (68–87) years, range 18–102, 416 (78%) aged ≥65 years, 189 (35%) aged ≥ 85 years; 315 (59%) female) were assessed in EMU over the four-month study period (Table 1, Fig. 1). Most patients were referred by their primary care practitioner (Table 1). Nearly half of patients used the dedicated EMU transport service at least once to reach the EMU with higher rates in older patients: 115 (51%) and 129 (68%) for 65–84 years and ≥ 85 years respectively (Table 1).

**Table 1:** Cohort descriptives including patient demographics, co-morbidity and markers of frailty by age group (< 65 years, 65–84 years and ≥ 85 years)

	Age group, years				
	All ***N = 533***	< 65 ***N = 117***	65–84 ***N = 227***	≥ 85 ***N = 189***	***p***
*Demographics & Living Arrangements*					
Age, mean/SD years	75.0/17.5	47.0/13.3	77.1/5.4	89.7/3.6	< 0.0001
Female	315 (59)	63 (54)	130 (57)	122 (65)	0.14
Living in own home	453 (86)	113 (97)	197 (88)	143 (78)	< 0.0001
Living alone	212 (40)	31 (27)	85 (38)	96 (51)	< 0.0001
Any care (formal, informal, or care home)	283 (54)	17 (15)	125 (55)	141 (77)	< 0.0001
Informal care (family, friends)	173 (32)	11 (9)	82 (36)	80 (42)	< 0.0001
Care package in own home	126 (24)	8 (7)	55 (24)	63 (33)	< 0.0001
Care home or supported living	71 (13)	3 (3)	27 (12)	41 (22)	< 0.0001
*Referrer and transport*					
GP	443 (83)	106 (91)	185 (81)	152 (80)	0.01
Paramedic	55 (10)	3 (3)	24 (11)	28 (15)	
Other	35 (7)	8 (7)	16 (7)	9 (5)	
Dedicated EMU transport required	261 (49)	17 (15)	115 (51)	129 (68)	< 0.0001
*Co-morbidities*					
Charlson Comorbidity Index > 3	395 (74)	18 (15)	190 (84)	187 (99)	< 0.0001
Medications > 7	243 (46)	26 (22)	129 (57)	88 (47)	< 0.0001
*Functional status*					
Barthel Index < 20	299 (56)	16 (14)	135 (59)	148 (78)	< 0.0001
Premorbid modified Rankin Scale > 2	193 (36)	7 (6)	91 (40)	95 (50)	< 0.0001
History of falls	143 (27)	2 (2)	60 (26)	81 (43)	< 0.0001
Visual impairment	65 (12)	2 (2)	29 (13)	34 (18)	< 0.0001
Hearing impairment	85 (16)	4 (3)	27 (12)	54 (29)	< 0.0001
Urinary incontinence^a^	91 (17)	6 (5)	35 (15)	50 (26)	< 0.0001
Faecal incontinence or stoma	42 (8)	3 (3)	24 (11)	15 (8)	0.03
*Cognitive status*					
Diagnosis of dementia	75 (14)	0 (0)	35 (15)	40 (21)	< 0.0001
*Nutrition*					
Poor nutritional status (MUST > 0)	105 (20)	9 (8)	50 (22)	46 (24)	< 0.0001
Patient’s perception of any weight loss	189 (35)	6 (5)	49 (22)	42 (22)	< 0.0001
*Other Frailty markers*					
Pressure sore risk (Braden Score < 19)	180 (34)	3 (3)	84 (38)	93 (50)	< 0.0001
Clinical impression of frailty	246 (46)	12 (10)	104 (46)	130 (70)	< 0.0001

^a^includes urinary catheters

### Residence, disability and dependency, co-morbidity and frailty

Although the majority of patients were resident in their own home, rates of disability and dependency were high especially in the oldest age group in whom 141 (77%) required some form of care input, 95 (50%) had mRS > 2 and 148 (78%) had Barthel < 20 (Table 1, Additional file 1: Fig. 1). Although rates were lower in those aged 65–84 years, they were nevertheless substantial: 125 (55%) requiring some form of care, 91 (40%) with mRS > 2 and 135 (59%) with Barthel < 20. As expected, patients aged < 65 years were much less likely to be dependent or disabled (Table 1, Additional file 1: Fig. 1).

In addition to dependency and disability, the cohort overall had a high burden of co-morbidity and pre-existing markers of frailty with substantial variation as expected by age (Table 1, Additional file 1: Figs. 1 and 2). Charlson co-morbidity index > 3 was present in 395 (74%) overall, and 243 (46%) had more than seven prescribed medications. In those aged ≥85 years, 81 (43%) had a history of falls, 50 (26%) had urinary incontinence or a long-term catheter, 72 (38%) had visual or hearing impairment, 40 (21%) had a dementia diagnosis. Other markers of frailty were also prevalent: 130 (70%) were judged to be frail by the assessing clinician, 46 (24%) had poor nutritional status and 93 (50%) were at risk of skin breakdown by Braden score (Table 1, Additional file 1: Fig. 2).

### Presenting complaints, illness severity and diagnoses

Older patients often presented with non-specific complaints including decreased mobility (122 (65%) of ≥ 85 years and 120 (53%) of 65–84 years), increased care needs (116 (61%) and 103 (45%)), falls (63 (33%) and 39 (17%)) or change in cognitive status (53 (28%) and 40 (18%)) whereas breathlessness was the most common presentation in younger patients (50 (43%) of < 65 years, Table 2). Other presenting complaints/problems were in keeping with an acute medical admissions unit and patients often reported multiple problems (see Additional file 1: Table 2). Notably, rates of severe illness, defined by SIRS> 1 or NEWS> 4, were broadly similar across age groups with overall rates of 148 (28%) and 102 (19%) respectively. In contrast, delirium affected 46 (24%) of ≥85 years versus only 1 (1%) of < 65 years with low AMTS in 94 (50%) versus 4 (3%, Table 2, Additional file 1: Fig. 2). The majority of patients received blood tests, x-rays and intravenous therapy although older patients were more likely to have existing medications stopped (63 (33%) of ≥ 85 years, 45 (20%) of 65–84 years, Table 2).

**Table 2:** Presenting complaint, illness severity, acute cognitive status, and diagnoses by age group (< 65 years, 65–84 years and ≥ 85 years)

	Age group, years				
	All ***N = 533***	< 65 ***N = 117***	65–84 ***N = 227***	≥ 85 ***N = 189***	***p***
*Presenting complaint, most frequent*^*a*^					
Decreased mobility	256 (48)	14 (12)	120 (53)	122 (65)	< 0.0001
Increased care needs	230 (43)	11 (9)	103 (45)	116 (61)	< 0.0001
Breathlessness	198 (37)	50 (43)	79 (35)	69 (37)	0.34
Falls	106 (20)	4 (3)	39 (17)	63 (33)	< 0.0001
Fatigue, weight loss, reduced oral intake	104 (20)	17 (15)	49 (22)	38 (20)	0.29
Abdominal symptoms (pain, bloating, diarrhoea, nausea, vomiting, constipation)	104 (20)	28 (24)	42 (19)	34 (18)	0.39
Confusion/altered behaviour	96 (18)	3 (3)	40 (18)	53 (28)	< 0.0001
Other respiratory symptoms (cough, wheeze, sore throat)	80 (15)	24 (21)	32 (14)	24 (13)	0.16
Chest pain/tachycardia/palpitations	57 (11)	20 (17)	27 (12)	10 (5)	0.004
*Illness severity*					
NEWS > 4	102 (19)	16 (14)	48 (22)	38 (20)	0.23
SIRS > 1	148 (28)	28 (24)	69 (30)	51 (27)	0.67
*Diagnosis, most frequent*^*a*^					
Bacterial Infection (LRTI>UTI > cellulitis)	214 (40)	47 (40)	89 (39)	78 (41)	0.91
Dehydration (clinical diagnosis)	163 (31)	18 (15)	73 (32)	72 (38)	< 0.0001
Anaemia	66 (12)	16 (14)	28 (12)	22 (12)	0.75
Electrolyte derangement	58 (11)	5 (4)	27 (12)	26 (14)	0.03
Heart Failure	57 (11)	3 (3)	21 (9)	33 (17)	< 0.0001
Acute Kidney Injury	51 (10)	5 (4)	24 (11)	22 (12)	0.15
*Acute cognitive assessment*					
Delirium	87 (16)	1 (1)	40 (18)	46 (24)	< 0.0001
AMTS < 9	160 (30)	4 (3)	62 (27)	94 (50)	< 0.0001
*Diagnostics and Interventions*					
Point-of-care blood test	444 (83)	89 (76)	193 (85)	162 (86)	0.06
Laboratory blood test	396 (74)	80 (68)	170 (75)	146 (77)	0.22
Any X-ray	302 (57)	48 (41)	130 (57)	124 (66)	< 0.0001
Intravenous treatment (e.g. antibiotics, blood, iron, or furosemide)	305 (57)	57 (49)	129 (57)	119 (63)	0.05
Oral medication (e.g. antibiotics, analgesia, end of life medications, laxatives)	241 (45)	48 (41)	105 (46)	88 (47)	0.59
Medication stopped	116 (22)	8 (7)	45 (20)	63 (33)	< 0.0001
Therapy input required	94 (18)	4 (3)	40 (18)	50 (26)	< 0.0001
*Ambulatory outcome*					
Remained ambulatory at 30 days^b^	315 (60)	96 (87)	128 (57)	91 (48)	< 0.0001
Ambulatory pathway after first assessment	381 (71)	109 (93)	153 (67)	119 (63)	< 0.0001
Admitted to hospital after first assessment	152 (29)	8 (7)	74 (33)	70 (37)	
Acute Hospital	47 (9)	5 (4)	25 (11)	17 (9)	
EMU Bed	90 (17)	3 (3)	42 (19)	45 (24)	
Other Community Hospital	15 (2)	0 (0)	7 (3)	8 (4)	
Unplanned admission within 30 days	58 (11)	8 (5)	23 (10)	27 (14)	
*Mortality*					
Death ≤ 30 days	31 (6)	1 (0)	14 (6)	16 (10)	0.03
Death ≤ 1 year^c^	138 (28)	7 (6)	57 (26)	74 (42)	< 0.0001

^a^Patients could have more than one presenting complaint or diagnosis

^b^For 8 patients, 30-day outcome was unavailable

^c^Data unavailable on 10 patients

*LRTI* lower respiratory tract infection, *UTI* urinary tract infection

Of the most common diagnoses, bacterial infection occurred in 214 (40%, of which 111 (52%) were respiratory) with similar rates across age groups. Dehydration and heart failure (both *p* < 0.0001), and to a lesser extent, electrolyte disturbance (*p* = 0.03), were more common in older patients but anaemia and acute kidney injury (AKI) showed no significant age-related variation (Table 2, Additional file 1: Table 3).

### Patient outcomes

**Fig. 2:**
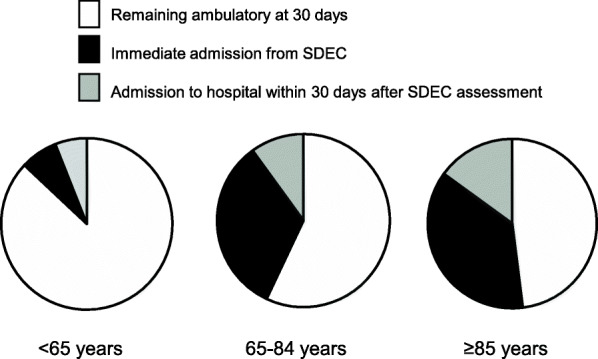
Proportion of patients remaining on an ambulatory pathway at 30 days by age group (< 65 years, 65–84 years and ≥ 85 years) versus hospital admission immediately ie directly from EMU assessment or within 30-days after initial EMU assessment

Overall, 210 (39%) of patients were admitted to hospital for bed-based care by 30-days (Fig. 1). The oldest patients were least likely to remain on an ambulatory pathway: 96 (87%) of those aged < 65 years remained ambulatory versus only 91 (48%) of those aged ≥ 85 years (*p* < 0.0001, Table 2, Fig. 2). Most of the 210 patients requiring hospital bed-based care were admitted immediately following first EMU assessment rather than subsequently: 152/210 (72%) versus 58 (28%, Table 2, Figs. 1 and 2). Immediate admission was usually to an EMU in-patient bed (90/152 (59%) with the majority of the remainder going to the regional acute hospital (OUHFT, Table 2). In the oldest patient group, 16 (10%) had died within 30 days and 74 (42%) by 1 year compared to 14 (6%) and 57 (26%) in those aged 65–84 years and 1 and 7 (6%) in those aged < 65 years (*p* < 0.0001, Table 2). Most deaths within 30-days (25/31) occurred in admitted patients (Fig. 1).

### Factors associated with admission for bed-based care

Factors associated with non-ambulatory status at 30 days are shown in Table 3 with adjustment for age and sex and with factors significant at the *p* < 0.001 level shown in bold (ie with the significance level corrected for the number of variables). Non-ambulatory patients were older than ambulatory patients (mean/sd age = 81.4/17.5 vs 71.5/18.6 years, *p* < 0.0001) and had more frailty and severe illness. There were associations with measures of disability/dependency including need for transport (OR = 2.84, 1.91–4.24, *p* < 0.0001), any care at home (OR = 2.17, 1.42–3.31, *p* < 0.0001), mRS > 2 (OR = 1.76, 1.48–2.09, *p* < 0.0001), referral with increased care needs (OR = 7.61, 4.94–11.74, *p* < 0.0001), and requirement for therapy input (4.32, 2.87–6.51, *p* < 0.0001). Strong associations (all *p* < 0.0001) were also seen for cognitive factors including delirium (OR = 17.54, 8.17–37.66), presentation with confusion or altered behaviour (OR = 7.04, 4.05–12.24), low AMTS (OR = 3.32, 2.17–5.08) and dementia (OR = 2.65, 1.56–4.50, *p* < 0.0001).

**Table 3:** Factors associated with ambulatory versus non-ambulatory status at 30 days (OR and *p* values shown adjusted for age and sex), bold values are those significant at the *p* = 0.001 level

	Ambulatory ***n*** = 315^**a**^	Admitted ***n*** = 210	OR	CI (95%)	***p***
*Demographics and Transport*					
Age (mean +/− SD)	71.5 (18.6)	81.4 (17.5)			
Female	187 (59.4)	124 (59.1)	0.87	0.60–1.23	0.45
** Transport required**	**115 (36.5)**	**145 (69.7)**	**2.84**	**1.91–4.24**	**< 0.0001**
*Co-morbidities*					
Charlson Score > 3	204 (64.8)	188 (89.5)	2.46	1.24–4.89	0.01
Number of medications> 7	133 (42.2)	108 (51.4)	1.14	0.79–1.64	0.50
*Physical Frailty markers*					
**Any care at home**	**135 (42.9)**	**144 (70.9)**	**2.17**	**1.42–3.31**	**< 0.0001**
**Modified Rankin Scale > 2**	**149 (47.3)**	**177 (84.3)**	**1.76**	**1.48–2.09**	**< 0.0001**
Barthel< 20	145 (46.0)	151 (71.9)	1.99	1.30–3.05	0.001
**History of falls**	**56 (17.8)**	**86 (41.3)**	**2.30**	**1.51–3.50**	**< 0.0001**
Visual impairment	27 (8.6)	36 (17.1)	1.74	1.01–3.01	0.05
Hearing impairment	36 (11.4)	48 (22.9)	1.57	0.95–2.57	0.08
Urinary incontinence^**b**^	45 (14.3)	67 (32.2)	2.03	1.28–3.21	0.002
**Faecal incontinence or stoma**	**17 (5.4)**	**40 (19.2)**	**3.44**	**1.85–6.41**	**< 0.0001**
MUST> 0	46 (14.6)	58 (27.6)	1.87	1.19–2.94	0.007
**Braden score < 19**	**62 (19.7)**	**115 (54.8)**	**3.62**	**2.38–5.50**	**< 0.0001**
Patient’s perception of any weight loss	41 (13.0)	56 (26.7)	1.74	1.18–2.55	0.005
**Therapy input required**	**58 (18.4)**	**117 (55.7)**	**4.32**	**2.87–6.51**	**< 0.0001**
** Clinician imp. of frailty**	**104 (33.0)**	**140 (67.3)**	**3.05**	**2.03–4.58**	**< 0.0001**
*Cognitive frailty markers*					
**Dementia**	**25 (7.9)**	**50 (24.0)**	**2.65**	**1.56–4.50**	**< 0.0001**
**Delirium**	**8 (2.5)**	**78 (37.1)**	**17.54**	**8.17–37.66**	**< 0.0001**
**AMTS < 9**	**56 (17.8)**	**104 (49.5)**	**3.32**	**2.17–5.08**	**< 0.0001**
*Presenting complaint*					
Shortness of breath	130 (41.3)	64 (30.8)	0.65	0.44–0.95	0.025
**Confusion/altered behaviour**	**19 (6.0)**	**77 (37.0)**	**7.04**	**4.05–12.24**	**< 0.0001**
**Decreased mobility**	**101 (32.1)**	**152 (73.1)**	**4.40**	**2.93–6.61**	**< 0.0001**
**Increased care needs**	**74 (23.5)**	**153 (73.6)**	**7.61**	**4.94–11.74**	**< 0.0001**
Falls	44 (14.0)	62 (29.8)	1.87	1.15–2.94	0.007
*Illness severity and diagnosis*					
**SIRS > 1**	**75 (23.8)**	**82 (39.0)**	**2.31**	**1.54–3.46**	**< 0.0001**
**NEWS > 4**	**42 (13.3)**	**59 (28.1)**	**2.47**	**1.56–3.93**	**< 0.0001**
**Any bacterial infection**	**106 (33.7)**	**104 (50.0)**	**2.09**	**1.43–3.04**	**< 0.0001**
**Clinical dehydration**	**66 (21.0)**	**95 (45.7)**	**2.66**	**1.79–3.95**	**< 0.0001**
**Anaemia**	**56 (17.8)**	**14 (6.7)**	**0.30**	**0.16–0.56**	**< 0.0001**
Electrolyte derangement	27 (8.6)	30 (14.3)	1.45	0.83–2.56	0.19
Heart failure	33 (10.5)	24 (11.4)	0.78	0.44–1.39	0.41
AKI	22 (7.0)	29 (13.9)	1.65	1.09–2.51	0.02
*Diagnostics*					
**Point-of-care blood test**	**245 (77.8)**	**193 (91.9)**	**3.05**	**1.71–5.44**	**< 0.0001**
Laboratory blood test	225 (71.4)	167 (79.5)	1.49	1.00–2.29	0.07
**Any X-ray**	**155 (49.2)**	**144 (69.2)**	**2.06**	**1.41–3.02**	**< 0.0001**
Intravenous treatment	161 (51.1)	139 (66.8)	1.74	1.19–2.53	0.004
Oral medication	133 (42.2)	106 (50.5)	1.39	0.96–2.00	0.08
Medication stopped	54 (17.1)	62 (29.5)	1.55	1.00–2.40	0.05
*Mortality*					
**Death < 30 days**	**6 (1.9)**	**25 (11.9)**	**5.60**	**2.23–14.09**	**< 0.0001**
**Death < 1 year**	**44 (14.0)**	**83 (39.5)**	**3.52**	**2.27–5.45**	**< 0.0001**

^a^30-day outcomes were unavailable for 8 patients. ^b^includes urinary catheters

Physical frailty markers were associated (all *p* < 0.0001) with admission for bed-based care including referral with decreased mobility (OR = 4.40, 2.93–6.61, *p* < 0.0001), faecal incontinence (OR = 3.44, 1.85–6.41, *p* < 0.0001), pressure sore risk (OR = 3.62, 2.38–5.50, *p* < 0.0001), clinical impression of frailty (OR = 3.05, 2.03–4.58, *p* < 0.0001) and history of falls (OR = 2.30, 1.51–3.50, *p* < 0.0001). Severe illness, whether measured by SIRS or NEWS, was also associated with non-ambulatory status at 30 days (OR = 2.31, 1.54–3.46 and OR = 2.47, 1.56–3.93, both *p* < 0.0001) as was bacterial infection (OR = 2.09, 1.43–3.04, *p* < 0.0001) and clinical dehydration (OR = 2.66, 1.79–3.95, *p* < 0.0001) but patients with anaemia were less likely to be admitted (OR = 0.30, 0.16–0.56, *p* < 0.0001).

155/192 (81%) of patients judged to be non-frail by the assessing clinician without severe illness (SIRS negative) remained ambulatory versus 19/69 (28%, *p* < 0.0001) of those judged to be frail who were severely ill (SIRS positive, Additional file 1: Fig. 3). Patients requiring hospital admission for bed-based care were much more likely to die within 30 days of initial EMU assessment (OR = 5.60, 2.23–14.09, *p* < 0.0001) and by 1 year (OR = 3.53, 2.27–5.45, *p* < 0.0001).

Comparing patients with immediate admission to hospital from the EMU (*N* = 152) with the 58 patients who were admitted later within the 30-days, showed that immediate admission was associated with referral with increased care needs, history of falls, need for therapy input, severe illness by NEWS, and delirium (Table 4).

**Table 4:** Factors associated with immediate versus later hospital admission (OR and p values shown adjusted for age and sex)

	Immediate admission *N = 152*	Later admission *N = 58*	OR	95% CI	*p*
Age, mean/sd	81.9/11.0	78.8/16.1	1.02	0.99–1.04	0.12
Sex, female	90 (59)	34 (59)	0.96	0.51–1.79	0.89
Referred with increased care needs	119 (78)	35 (60)	2.27	1.14–4.51	0.02
History of falls	73 (48)	13 (22)	3.02	1.48–6.16	0.002
Need for therapy input	93 (61)	24 (41)	2.12	1.13–3.96	0.02
NEWS> 4	51 (34)	8 (14)	3.18	1.40–7.23	0.006
Delirium	67 (44)	11 (19)	3.18	1.50–6.71	0.002

Numbers are n (%) unless otherwise specified

Multivariable logistic regression to determine the independent associates of any bed based care within 30-days gave similar results whether SIRS or NEWS was used to define illness severity (Table 5, Additional file 1: Table 4). Delirium was the factor most strongly associated (OR = 11.93 (3.70–38.50, *p* < 0.0001 using SIRS to define illness severity in the model). Other independent factors included the need for the dedicated EMU-transport service, referral with decreased functional ability and increased care needs, and severe illness (Table 5, Additional file 1: Table 4). There were also trends towards associations with vision impairment, history of falls and AKI.

**Table 5:** Factors independently associated with any admission to bed-based care within 30-days, for the cohort overall and after exclusion of patients with delirium

Factor	Using SIRS all patients		Using NEWS all patients		Using SIRS patients without delirium		Using NEWS patients without delirium	
	OR (95% CI)	***p***	OR (95% CI)	***p***	OR (95% CI)	***p***	OR (95% CI)	***p***
EMU transport required	**1.74 (1.04–2.92)**	**0.04**	**1.76 (1.05–2.96)**	**0.03**	**2.01 (1.17–3.45)**	**0.01**	**1.99 (1.15–3.44)**	**0.01**
History of falls	1.85 (0.94–3.66)	0.08	1.79 (0.90–3.54)	0.10	1.27 (0.67–2.38)	0.46	1.26 (0.67–2.41)	0.45
Referred with decreased mobility	**1.91 (1.03–3.53)**	**0.04**	**1.97 (1.06–3.65)**	**0.03**	**2.18 (1.14–4.18)**	**0.02**	**2.27 (1.18–4.35)**	**0.01**
Referred with increased care needs	**3.26 (1.71–6.23)**	**< 0.0001**	**3.17 (1.65–6.06)**	**0.001**	**2.90 (1.48–5.68)**	**0.002**	**2.88 (1.47–5.64)**	**0.002**
Vision impaired	1.83 (0.94–3.58)	0.08	1.89 (0.96–3.71)	0.06	1.91 (0.94–3.89)	0.07	1.81 (0.89–3.70)	0.10
Delirium	**11.93 (3.70–38.50)**	**< 0.0001**	**10.97 (3.36–35.76)**	**< 0.0001**	**–**	**–**	**–**	**–**
Bacterial infection	1.41 (0.87–2.31)	0.17	1.57 (0.96–2.57)	0.07	1.34 (0.80–2.24)	0.26	1.49 (0.89–2.49)	0.13
AKI	1.64 (0.93–2.87)	0.09	1.66 (0.93–2.96)	0.09	1.65 (0.92–2.97)	0.10	1.69 (0.93–3.09)	0.09
SIRS, per point	**1.48 (1.17–1.87)**	**0.001**	**–**	**–**	**1.52 (1.19–1.94)**	**0.001**	**–**	**–**
NEWS, per point			**1.17 (1.05–1.30)**	**0.003**			**1.20 (1.08–1.34)**	**0.001**

Models included age, sex, transport required, care required at home, referred with decreased mobility, referred with increased confusion/altered behaviour, referred with reduced mobility, history of falls, vision impairment, hearing impairment, dementia diagnosis, urinary incontinence, faecal incontinence, dehydration, anaemia, bacterial infection, delirium, SIRS/NEWS. The model did not include factors not routinely acquired at first assessment in urgent care settings (Barthel, mRS, MUST, Braden, Charlson index). Table shows factors significant at *p* < 0.05 in at least one of the models, or factors showing a trend to an association (*p* ≤ 0.10)

## Discussion

In this unselected cohort from a multidisciplinary SDEC unit with a predominance of older people, any bed based care at 30 days occurred in one half of those over 65 years, and was associated with markers of frailty and illness severity/acuity. The highest rates of admission were seen in patients with severe illness occurring in the context of frailty, particularly cognitive frailty (delirium).

To our knowledge, this is the first study of an SDEC service with inclusive referral criteria designed around the needs of older patients. Our cohort showed a wide range of presenting complaints and diagnoses in line with what might be expected in an acute medical admissions unit. Non-specific presentations were common including changes in functional status, increased care needs and confusion or altered behavior. Severe illness affected around one quarter of patients and intravenous therapy was required in the majority. In contrast, in previous studies of ambulatory emergency care for older patients chosen by a geriatrician for comprehensive geriatric assessment/multidisciplinary ambulatory care, falls or syncope were often the most common reasons for referral [14], consistent with a high level of patient selection, and no data were given on illness severity/acuity [14, 15].

We did not have data on specific frailty scores such as the clinical frailty scale [31] since such scores were not a routine part of patient assessment on the EMU. However, detailed multidisciplinary assessment was undertaken covering domains generally considered to be integral to a comprehensive geriatric assessment [9]. Data from these assessments showed a high prevalence of frailty markers in the cohort: nearly half were considered frail by the treating clinician, frailty syndromes including falls, incontinence, visual or hearing deficits and cognitive impairment were common, and many patients were at risk of malnutrition or pressure sores. Multimorbidity (Charlson index> 3) affected three quarters of the patients overall and the majority of older patients required some form of care at home and had some degree of disability or dependency.

Frailty markers were associated with admission for bed-based care and two thirds of those considered frail by the assessing clinician were hospitalized. Admission was more likely if the frail person was also severely ill or delirious (< 3% of those with delirium avoided hospital admission) but rates of admission were nevertheless higher in frail patients without severe illness than in non-frail patients who were severely ill. Although delirium was strongly associated with admission, other factors associated with admission were qualitatively similar in those with and without delirium.

Our findings are in keeping with data from the Emergency Department/medical admissions setting in which patients with frailty were less likely to be discharged immediately or to have short (< 72 h) admissions, and only 23–40% of non-admitted patients were frail [13]. Although multi-morbidity as defined by the Charlson index was more common as expected in the older patients in our study, associations with admission for bed-based care were relatively weak and did not reach significance after Bonferroni correction, or in multivariable analyses. This illustrates the fact that multi-morbidity is not synonymous with frailty [2]. Similarly, although polypharmacy was common, there was no association between number of prescribed medications and hospital admission.

Although illness severity was associated with bed-based care, the proportion varied according to the method of illness severity assessment: a quarter of those classed as severely ill by SIRS [28] and around an eighth by NEWS [29] avoided hospital admission. The discrepancy between the two scores may be accounted for by the accepted cut-offs used to define illness severity and the inclusion of more vital sign variables in NEWS including “reduced level of consciousness”, the latter resulting in higher NEWS in those with hypoactive delirium. Since patients with delirium were more likely to be admitted, this partly explains why the number of patients remaining ambulatory with high NEWS was relatively lower. Looking at specific medical diagnoses, anaemia was associated with remaining on an ambulatory path whereas bacterial infection was more likely to result in admission. Bacterial infection was likely associated with more severe illness but the trend towards an independent association in multivariable analyses suggests that there may be other factors underlying admission in those with infection.

Our findings suggest that most of the risk of admission for bed-based care from an SDEC setting is conferred by a few factors capturing physical and cognitive frailty, change in functional and or cognitive status, and the presence of illness acuity. These factors were more associated with immediate versus later hospital admission after SDEC assessment. In particular, acute change in cognition as defined by delirium, rather than established dementia or cognitive impairment in the absence of delirium, appears important.

Our findings may help in triaging of patients to the appropriate care facility even prior to SDEC referral as well as in the SDEC itself. Currently, there are no risk prediction models to aid decision making in the SDEC environment but models have been developed in the acute medicine/emergency setting. The AMB [32] and Glasgow Admission Prediction Score (GAPS) [33] were developed to predict same day discharge and include measures of illness severity. The AMB score also includes “access to personal/public transport” and absence of “acute confusion” [32]. Both require information on previous hospital admissions, which may not be easily available, but not other measures of frailty. The recently developed Sydney Triage to Admission Risk Tool (START+) does include a physical frailty measure but this requires collection using a specific questionnaire [34]. No available scores to our knowledge include delirium, despite its importance in predicting hospital admission, probably because the development cohorts did not contain detailed data on cognitive frailty syndromes.

Strengths of our study include the use of data from a prospective consecutive cohort with no exclusion criteria, careful and detailed patient phenotyping for cognitive and physical frailty using established methodology [19], and importantly, measurement of illness severity/acuity. We also ascertained ambulatory status up to 30-days after first EMU assessment rather than only on the day of first assessment. Limitations of our study include first, that our study was from a single centre and other SDEC units will have different settings and operational criteria. However, the EMU case-mix was broadly representative of current real world emergency acute internal/geriatric medicine practice and was free of selection bias in contrast to other studies of pre-selected patients likely to be suitable for ambulatory care [14, 15]. Second, we did not have data on specific frailty scores but patients were assessed in detail for markers of both cognitive and physical frailty. In addition, existing frailty scales may not be valid in acute hospital settings [35] and do not contain detailed cognitive assessments including for delirium. Third, determining the specific reasons why patients were admitted after SDEC assessment was beyond the scope of our study. However, the strong independent associations between hospital admission, and increased care needs, delirium and severe illness suggests that need for care is a key factor together with access to physiologic monitoring and treatment escalation. Our findings will inform future studies around interventions to enhance ambulatory pathways to reduce hospital admission as well as service development.

## Conclusions

Our study shows that, even in an SDEC with a multidisciplinary approach incorporating comprehensive geriatric assessment, around three-quarters of older frail patients with severe acute illness as judged by SIRS, were admitted for bed-based care. Expansion of alternatives to acute bed-based care should recognize the dependency and acuity of patients and should not replace continued provision of high quality inpatient frailty services. Further studies are required to understand the key components of hospital bed-based care that need to be replicated by models delivering acute frailty care closer to home, and the feasibility, cost-effectiveness and patient/carer acceptability of such models.

## Supplementary Information

**Additional file 1: Table 1.** Missing Data. **Table 2.** Presenting complaints. **Table 3.** Diagnoses. **Table 4.** Factors independently associated with any admission to bed-based care within 30-days for the cohort overall and after exclusion of patients with delirium: multivariable analysis with all factors significant in univariable analyses including scores not routinely collected at first patient assessment in most acute care settings. **Figure 1.** Bar graph showing living arrangements, requirement for Same Day Emergency Care (SDEC) dedicated transport, comorbidities and dependency (mRS>2) for the cohort by age group (black bars=>85 years, grey bars=65-84 years, white bars=<65 years). **Figure 2.** Markers of physical (left hand graph) and cognitive (right hand graph) frailty by age group (black bars=>85 years, grey bars=65-84 years, white bars=<65 years). **Figure 3.** Percentage of patients admitted to hospital within 30-days of Same Day Emergency Care (SDEC) assessment in not frail (left hand graph) and frail (clinician’s impression of frailty – right hand graph) by presence or absence of severe illness as defined by the systemic inflammatory response syndrome (SIRS>2).

## Data Availability

Applications for access to data should be made to Professor Sarah Pendlebury (sarah.pendlebury@ndcn.ox.ac.uk) although access to researchers outside the University of Oxford and Oxford University Hospitals NHS Trust is restricted under the terms of the ethics approval.

## Abbreviations

AKI: Acute kidney injury
AMTS: Abbreviated Mental Test Score
CAM: Confusion Assessment Method
EMU: Emergency Medicine Unit
GAPS: Glasgow Admission Prediction Score
GP: General Practitioner
ORCHARD: Oxford Cognitive Co-morbidity and Frailty Ageing Research Database
OUHFT: Oxford University Hospitals NHS Foundation Trust
mRS: Modified Rankin Score
MUST: Malnutrition Universal Screening Tool
NEWS: National Early Warning Score
REC: Research ethics committee
SDEC: Same Day Emergency Care
SIRS: Systemic inflammatory response syndrome
START: Sydney Triage to Admission Risk Tool
