# Supplementary material for: Factors associated with admission to bed-based care: observational prospective cohort study in a multidisciplinary same day emergency care unit (SDEC)
Source: BMC Geriatr. 2021 Jan 6;21:8. doi: 10.1186/s12877-020-01942-9 (PMC7788859; doi:10.1186/s12877-020-01942-9)
Supplement: Supplementary file 1 — Additional file 1: Table 1. Missing Data. Table 2. Presenting complaints. Table 3. Diagnoses. Table 4. Factors independently associated with any admission to bed-based care within 30-days for the cohort overall and after exclusion of patients with delirium: multivariable analysis with all factors significant in univariable analyses including scores not routinely collected at first patient assessment in most acute care settings. Figure 1. Bar graph showing living arrangements, requirement for Same Day Emergency Care (SDEC) dedicated transport, comorbidities and dependency (mRS>2) for the cohort by age group (black bars=>85 years, grey bars=65-84 years, white bars=<65 years). Figure 2. Markers of physical (left hand graph) and cognitive (right hand graph) frailty by age group (black bars=>85 years, grey bars=65-84 years, white bars=<65 years). Figure 3. Percentage of patients admitted to hospital within 30-days of Same Day Emergency Care (SDEC) assessment in not frail (left hand graph) and frail (clinician’s impression of frailty – right hand graph) by presence or absence of severe illness as defined by the systemic inflammatory response syndrome (SIRS>2). [file 12877_2020_1942_MOESM1_ESM.docx]

**Additional file**

**Factors associated with admission to bed-based care: observational prospective cohort study in a multidisciplinary same day emergency care unit (SDEC)**

Tania CN Elias^1,2^, Jordan Bowen, ^2^ Royah Hassanzadeh,^2^ Daniel S Lasserson,^3,4^ Sarah T Pendlebury^1,2,5^

^1^Wolfson Centre for Prevention of Stroke and Dementia, Nuffield Department of Clinical Neurosciences, John Radcliffe Hospital, and the University of Oxford.

^2^Departments of Acute Internal Medicine and Geratology, Oxford University Hospitals NHS Foundation Trust, John Radcliffe Hospital, OX3 9DU.

^3^PIONEER Health Data Research Hub, Institute for Applied Health Research, University of Birmingham, B15 2TT

^4^Department of Acute Medicine, City Hospital, Sandwell and West Birmingham Hospitals NHS Trust, B18 7QH

^5^NIHR Oxford Biomedical Research Centre, John Radcliffe Hospital, OX3 9DU.

Correspondence to:

Sarah Pendlebury

Centre for Prevention of Stroke and Dementia

Wolfson Building

John Radcliffe Hospital

Oxford OX3 9DU

Email: [sarah.pendlebury@ndcn.ox.ac.uk](mailto:sarah.pendlebury@ndcn.ox.ac.uk)

**Table 1: Missing Data**

Referrer not recorded in 1 patient (n=532)

Assessor not recorded in 5 patients (n=528)

Usual place of residence not recorded in 9 patients from community or acute hospitals (n=524)

Living alone not recorded in 5 patients (n=528)

Care arrangements not recorded in 6 patients (n=527)

Medications not recorded in 2 patients (n=531)

Sensory impairment not recorded in 4 patients (n=531)

MUST not recorded in 4 patients (n=529)

Braden not recorded in 10 patients (n=523)

Clinical impression of frailty not given in 2 (n=531)

>1 components of NEWS were unavailable in 10 patients (n=523)

30-day data missing in 10 patients who lived out of area and so could not be followed up (n=523)

SIRS was incomplete for 111 patients (missing white cell count component), and these were imputed to have ‘normal’ white cell count levels.

AMTS was not recorded in 176 (under 75 years and not felt to be necessary, or a small number unable or refused). If deemed not necessary, imputed to score of ‘10’. If unable or refused, imputed to score of ‘<9’.

Mortality data excludes 12 patients (out of area) and 20 repeat attendances (n=501).

**Table 2. Presenting complaints**

| Presenting Complaint | N (%) |
| --- | --- |
| Decreased mobility | 256 (48) |
| Increased care needs | 231 (43) |
| Breathlessness | 198 (37) |
| Falls | 106 (20) |
| Abdominal symptoms (pain, bloating, diarrhoea, nausea, vomiting, constipation) | 104 (20) |
| Fatigue, weight loss, reduced oral intake | 104 (20) |
| Confusion/ altered behaviour | 96 (18) |
| Other respiratory (cough, wheeze, sore throat) | 80 (15) |
| Chest pain, tachycardia or palpitations | 57 (11) |
| Pain (back, hip/groin, shoulder, knee, wrist, toe, facial) | 57 (11) |
| Symptomatic anaemia | 35 (7) |
| Urinary symptoms (dysuria, catheter problems, retention) | 34 (6) |
| Bilateral leg swelling | 34 (6) |
| Fever | 30 (6) |
| Rash/erythema (incl face) | 26 (5) |
| Biochemical derangement (electrolytes, renal function, LFTs) | 23 (4) |
| Dizziness / unsteadiness | 20 (4) |
| Unilateral swelling | 18 (3) |
| Other neurological (seizure, headache, tremor, visual symptoms, vertigo, tinnitus) | 15 (3) |
| Drowsiness | 11 (2) |
| Leg weakness | 10 (2) |
| Collapse | 9 (2) |
| Glycaemic derangement | 7 (1) |
| Rectal bleed / malaena | 4 (1) |
| Anxiety / low mood | 4 (1) |

**Table 3. Diagnoses**

| Diagnosis | N (%) |
| --- | --- |
| Any bacterial infection | 214 (40) |
| Pneumonia/LRTI | 111 (21) |
| Anaemia | 71 (13) |
| Electrolyte derangement (Na, K, Ca, Mg) | 58 (11) |
| Heart Failure | 57 (11) |
| Dehydration | 55 (10) |
| Urinary tract infection | 54 (10) |
| Acute Kidney Injury | 51 (10) |
| Adverse drug reaction | 47 (9) |
| Asthma/COPD/exacerbation other chronic lung disease | 38 (7) |
| Cellulitis | 35 (7) |
| Musculoskeletal pain (MSK / disc / OA / fragility fracture) | 35 (7) |
| Constipation | 34 (6) |
| Atrial fibrillation / arrhythmia | 24 (5) |
| Depression or anxiety | 20 (4) |
| Orthostatic hypotension | 18 (3) |
| Progression dementia or symptoms due to severe dementia | 17 (3) |
| Cancer symptoms (new or old) | 17 (3) |
| Other (biliary, sinus, endocarditis, bone, unknown) | 17 (3) |
| Non-infective diarrhoea | 16 (3) |
| GORD / Gastritis / dyspepsia | 12 (2) |
| Urinary retention / obstruction | 11 (2) |
| Pulmonary Embolism | 11 (2) |
| Skin rash | 11 (2) |
| Infective diarrhoea | 10 (2) |
| Viral infection | 10 (2) |
| Viral infection | 10 (2) |
| Surgical abdomen (bowel obstruction, ischaemic bowel, pancreatitis, diverticulitis) or urological surgical problem (ureteric colic) or vascular surgical problem (AAA) | 10 (2) |
| Parkinson's disease | 9 (2) |
| Cardiac chest pain (angina, ACS) | 9 (2) |
| Gout | 8 (2) |
| Diabetic glycaemic complication | 8 (2) |
| Seizure, muscular dystrophy, periph neuropathy, BIH | 7 (1) |
| Gastrointestinal bleed | 6 (1) |
| BPPV / labyrinthitis | 5 (1) |
| Pleural effusion | 4 (1) |
| Terminal illness | 4 (1) |
| Alcohol excess or withdrawal | 4 (1) |
| Stroke | 4 (1) |
| Leg ulcers | 4 (1) |
| Carer strain | 4 (1) |
| Haemothorax/ pneumothorax | 3 (1) |
| Thyroid or other endocrine (Addison's) disorder | 3 (1) |
| Gallstone disease | 3 (1) |
| Soft tissue injury | 3 (1) |
| DVT | 3 (1) |
| Pericarditis | 2 (0) |
| Korsakoff's / hepatic encephalopathy | 2 (0) |
| GCA / PMR / reactive arthritis | 2 (0) |
| Pleurisy | 2 (0) |
| Dysphagia | 1 (0) |
| Head injury | 1 (0) |
| Anaphylaxis | 1 (0) |
| Pregnancy-related symptoms | 1 (0) |
| Vasovagal syncope | 1 (0) |

Table 4. Factors independently associated with any admission to bed-based care within 30-days for the cohort overall and after exclusion of patients with delirium: multivariable analysis with all factors significant in univariable analyses including scores not routinely collected at first patient assessment in most acute care settings.

|  | Using SIRS  all patients | | Using NEWS  all patients | | Using SIRS  patients without delirium | | Using NEWS  patients without delirium | |
| --- | --- | --- | --- | --- | --- | --- | --- | --- |
| Factor | **OR (95% CI)** | **p** | **OR (95% CI)** | **p** | **OR (95% CI)** | **p** | **OR (95% CI)** | **p** |
| Transport required | **1.92 (1.13-3.27)** | **0.02** | **1.90 (1.11-3.24)** | **0.02** | **2.26 (1.28-3.97)** | **0.005** | **2.21 (1.25-3.90)** | **0.006** |
| Referred with decreased mobility | **1.85 (0.97-3.53)** | **0.06** | **1.89 (0.99-3.59)** | **0.05** | **2.35 (1.18-4.67)** | **0.02** | **2.40 (1.21-4.76)** | **0.01** |
| Referred with increased care needs | **3.08 (1.55-6.12)** | **0.001** | **3.03 (1.53-6.00)** | **0.001** | **2.98 (1.46-6.09)** | **0.003** | **2.88 (1.41-5.87)** | **0.004** |
| Vision impaired | 1.83 (0.90-3.73) | 0.10 | 1.68 (0.83-3.42) | 0.15 | 2.02 (0.94-4.34) | 0.07 | 1.79 (0.83-3.86) | 0.14 |
| Poor nutritional status | **2.07 (1.13-3.79)** | **0.02** | **2.03 (1.11-3.75)** | **0.02** | **2.45 (1.29-4.65)** | **0.006** | **2.37 (1.24-4.53)** | **0.009** |
| At risk of pressure sores | 1.78 (0.96-3.30) | 0.07 | 1.86 (1.00-3.46) | 0.05 | 1.81 (0.94-3.46) | 0.07 | 1.94 (1.00-3.74) | 0.05 |
| Delirium | **11.28 (3.07-41.44)** | **<0.0001** | **10.40 (2.88-37.59)** | **<0.0001** | **-** | **-** | **-** | **-** |
| Bacterial infection | 1.42 (0.86-2.35) | 0.17 | 1.59 (0.96-2.63) | 0.07 | 1.41 (0.83-2.39) | 0.21 | 1.54 (0.91-2.63) | 0.11 |
| SIRS, per point | **1.46 (1.15-1.87)** | **0.002** |  |  | **1.55 (1.20-2.00)** | **0.001** |  |  |
| NEWS, per point | **-** | **-** | **1.17 (1.05-1.30)** | **0.005** | **-** | **-** | **1.21 (1.08-1.36)** | **0.001** |

Models included age, sex, transport required, care required at home, referred with decreased mobility, referred with increased confusion/altered behaviour, referred with reduced mobility, referred with fall, referred with shortness of breath, history of falls, vision impairment, hearing impairment, dementia diagnosis, urinary incontinence, faecal incontinence, pressure sore risk (Braden), nutritional status (MUST), Charlson index, dehydration, anaemia, bacterial infection, delirium, AMTS, SIRS/NEWS. Table shows factors significant at p<0.05 in at least one of the models, or factors showing a trend to an association (p<0.10).

**Additional file Figure Legends**

Figure 1. Bar graph showing living arrangements, requirement for Same Day Emergency Care (SDEC) dedicated transport, comorbidities and dependency (mRS>2) for the cohort by age group (black bars=>85 years, grey bars=65-84 years, white bars=<65 years).

Figure 2. Markers of physical (left hand graph) and cognitive (right hand graph) frailty by age group (black bars=>85 years, grey bars=65-84 years, white bars=<65 years).

Figure 3. Percentage of patients admitted to hospital within 30-days of Same Day Emergency Care (SDEC) assessment in not frail (left hand graph) and frail (clinician’s impression of frailty – right hand graph) by presence or absence of severe illness as defined by the systemic inflammatory response syndrome (SIRS>2).


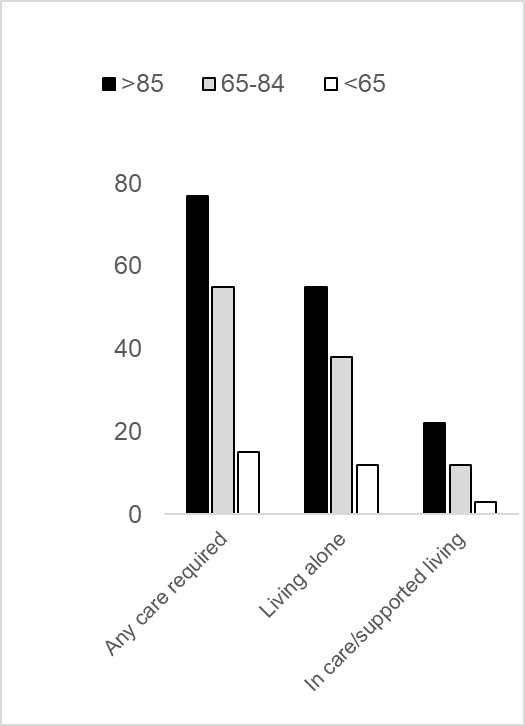

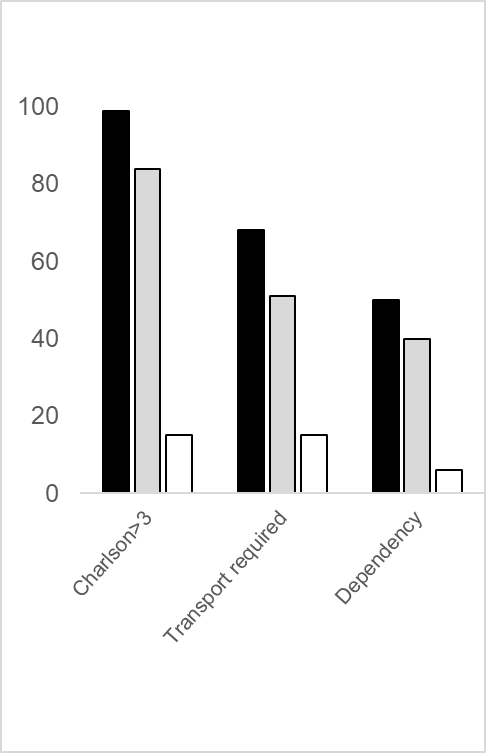


Percentage of cohort

Figure 1: Living arrangements, requirement for EMU dedicated transport, comorbidities and dependency (mRS>2) for the cohort by age group.


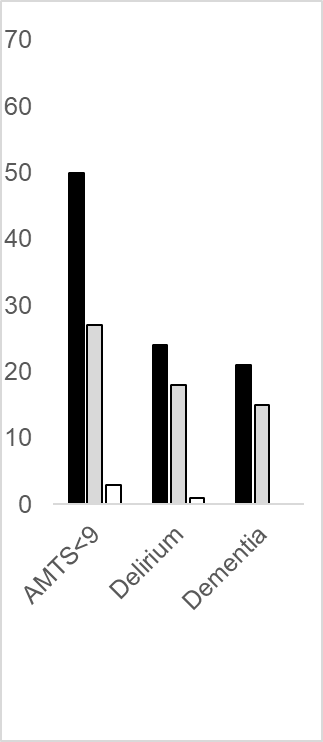

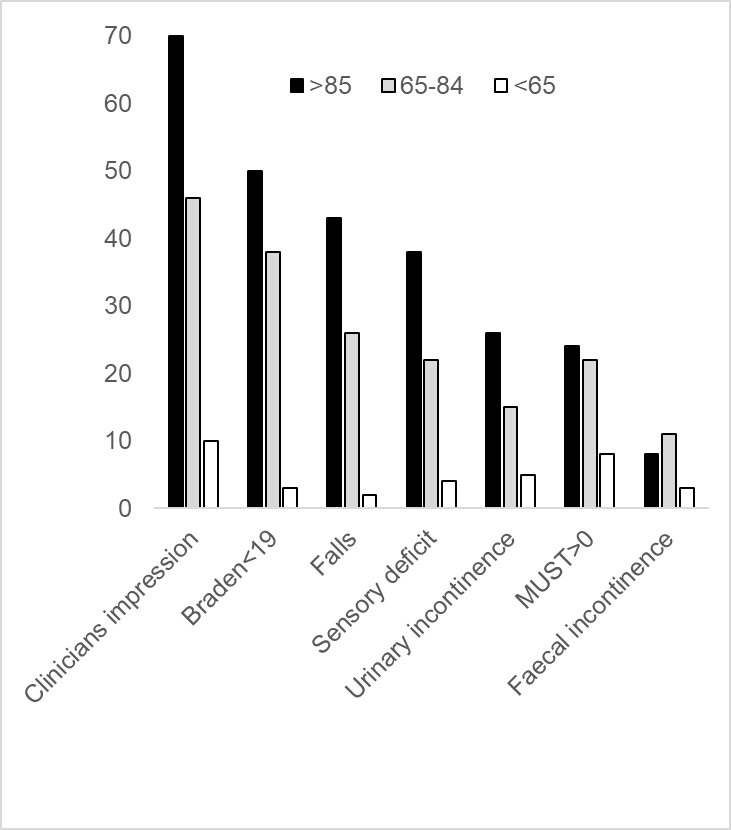


Percentage with frailty syndrome

Appendix Figure 2

Markers of physical and cognitive frailty by age group (<65 years, 65-84 years and >85 years).

Figure 2: Markers of physical and cognitive frailty by age group (<65 years, 65-84 years and >85 years).


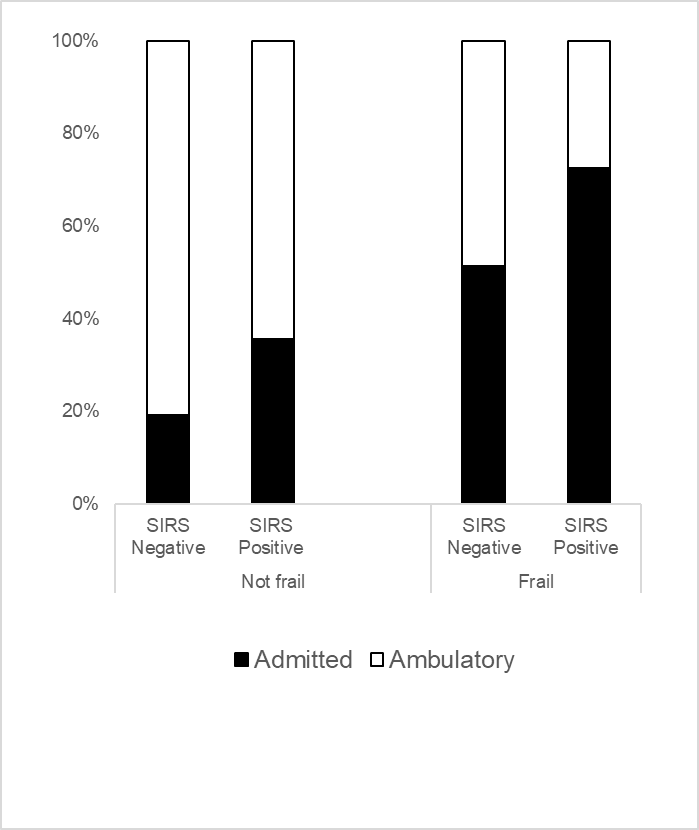


Percentage of patients

Not frail Frail

Figure 3: Percentage of patients admitted to hospital within 30-days of SDEC assessment in not frail and frail (clinician’s impression of frailty) groups by presence of severe illness (SIRS).

Appendix Figure 1

Living arrangements, requirement for EMU dedicated transport, comorbidities and dependency (mRS>2) for the cohort by age group
